# Efficient CO_2_ fixation by surface *Prochlorococcus* in the Atlantic Ocean

**DOI:** 10.1038/ismej.2014.56

**Published:** 2014-04-24

**Authors:** Manuela Hartmann, Paola Gomez-Pereira, Carolina Grob, Martin Ostrowski, David J Scanlan, Mikhail V Zubkov

**Affiliations:** 1 Ocean Biogeochemistry and Ecosystems Research Group, National Oceanography Centre, Southampton, UK; 2 School of Life Sciences, University of Warwick, Coventry, UK

**Keywords:** primary production, chlorophyll autofluorescence, flow cytometric cell sorting, ^14^C radiolabelling

## Abstract

Nearly half of the Earth's surface is covered by the ocean populated by the most abundant photosynthetic organisms on the planet—*Prochlorococcus* cyanobacteria. However, in the oligotrophic open ocean, the majority of their cells in the top half of the photic layer have levels of photosynthetic pigmentation barely detectable by flow cytometry, suggesting low efficiency of CO_2_ fixation compared with other phytoplankton living in the same waters. To test the latter assumption, CO_2_ fixation rates of flow cytometrically sorted ^14^C-labelled phytoplankton cells were directly compared in surface waters of the open Atlantic Ocean (30°S to 30°N). CO_2_ fixation rates of *Prochlorococcus* are at least 1.5–2.0 times higher than CO_2_ fixation rates of the smallest plastidic protists and *Synechococcus* cyanobacteria when normalised to photosynthetic pigmentation assessed using cellular red autofluorescence. Therefore, our data indicate that in oligotrophic oceanic surface waters, pigment minimisation allows *Prochlorococcus* cells to harvest plentiful sunlight more effectively than other phytoplankton.

## Introduction

Oceanic phytoplankton constitute only a minute fraction of the photosynthetic biomass on Earth (Falkowski, 2012), but they contribute almost half of the yearly global net primary production (Falkowski *et al.*, 1998; Field *et al.*, 1998; Behrenfeld *et al.*, 2001). *Prochlorococcus* (*Pro*) cyanobacteria are the most abundant phytoplankton in the ocean, inhabiting nutrient-depleted environments from the equator up to 40–50° of latitude (Campbell and Vaulot, 1993; Zubkov *et al.*, 2000). However, in surface waters the cellular pigment concentration of *Pro* is very low (∼0.1 fg divinyl chlorophyll per cell), and consequently their carbon to chlorophyll ratios are very high (93.3–122 mg C per mg chlorophyll-*a*) in comparison with *Synechococcus* cyanobacteria and small picoeukaryotes (38–58 mg C per mg chlorophyll-*a*) (Veldhuis and Kraay, 2004; Bouman *et al.*, 2006).

From their discovery onwards, *Pro* cells were almost exclusively enumerated by flow cytometry using their red autofluorescence and small size for identification (Chisholm *et al.*, 1988; Partensky *et al.*, 1999). Cellular red autofluorescence and chlorophyll pigment content are directly linked (Moore *et al.*, 1995; Cavender-Bares *et al.*, 1999; Dusenberry *et al.*, 2001), although the relation may not always be linear and can be affected by pigment ‘packaging' or variations in accessory pigments predominantly for larger, more heterogeneous cells (Sosik *et al.*, 1989). Low red autofluorescence of *Pro* cells hampered their flow cytometric enumeration (Olson *et al.*, 1990; Dusenberry and Frankel, 1994) and technical improvements such as tightening of the beam spot, broadening of laser excitation, the introduction of quartz flow cells with integrated lenses as well as the reduction of the sheath pressure were used to detect low pigmented *Pro* cells (Olson *et al.*, 1990; Cavender-Bares *et al.*, 1998). However, in the upper 40–80 m of stratified oligotrophic oceanic waters, it has remained a challenge to enumerate *Pro* cells unambiguously using their red autofluorescence (Chisholm *et al.*, 1988; Zubkov *et al.*, 1998; Partensky *et al.*, 1999; Ting *et al.*, 2002; Malmstrom *et al.*, 2010). In such surface waters, the flow cytometrically visible *Pro* population is typically shaped in the form of a ‘rising sun' emerging out of the background noise on a scatter plot of red autofluorescence (>650 nm) vs 90° side light scatter (Charles *et al.*, 2005 and Supplementary Figure S1). In some studies, an extrapolation based on the visible part of the *Pro* population was applied to correct for the missing part (see, for example, Partensky *et al.*, 1996). Nucleic acid staining of bacterioplankton was proposed as an alternative solution to the *Pro* cell detection problem (Zubkov *et al.*, 2000). Later molecular identification confirmed that a distinct population observed following nucleic acid staining consists mainly of *Pro* cells (Zubkov *et al.*, 2007; Mary *et al.*, 2008; Gomez-Pereira *et al.*, 2013). Direct comparison of the two approaches for *Pro* enumeration, that is, using red autofluorescence or cellular DNA-content/light scatter for identification, showed that in surface waters, up to a half of the *Pro* cells are unaccounted when red autofluorescence is used as the sole identifier (Zubkov *et al.*, 2000).

High irradiance and resulting bleaching of photosynthetic pigments alongside potential nutrient-limitation in surface waters of oceanic subtropical gyres are common explanations for almost colourless *Pro* cells in surface ocean waters. Because in deeper (>80 m) nutrient-replete but less illuminated parts of the water column, *Pro* pigmentation is much more intense (Partensky *et al.*, 1999), the CO_2_ uptake studies have focussed on *Pro* living in deeper waters (Chisholm *et al.*, 1988; Li, 1994).

We put to the test two alternative explanations that account for the extremely low photopigmentation of *Pro* cells in the nutrient-depleted surface waters: (1) pigment levels are decreased as a result of the combined effects of high irradiance and nutrient limitation that diminishes capacity of the cells to cope with this stress, or (2) constitutively low cellular levels of photosynthetically active pigmentation are adequately physiologically balanced for these environmental conditions. In the former case, *Pro* red autofluorescence-normalised CO_2_ fixation should be low compared with other phytoplankton, whereas red autofluorescence-normalised CO_2_ fixation of *Pro* cells will be comparable with, or higher than, red autofluorescence-normalised CO_2_ fixation by other phytoplankton cells if the latter were true.

Here, we present direct experimental evidence that red autofluorescence-normalised CO_2_ fixation of surface *Pro* is high compared with the smallest eukaryotic phytoplankton and *Synechococcus* cyanobacteria. These results demonstrate that the dim red autofluorescence of surface *Pro* does not prevent them attaining high CO_2_ fixation rates across the Atlantic Ocean.

## Materials and methods

### Sampling

Pre-dawn seawater samples were collected from 20 m depth in 20 l Niskin (Miami, FL, USA) bottles attached to a standard conductivity–temperature–depth profiler on the 20th cruise of the Atlantic Meridional Transect programme aboard the UK Royal Research Ship *James Cook* in October–November 2010 (Supplementary Figure S2). Seawater content of the entire Niskin bottle was decanted into an acid-rinsed polycarbonate carboy. To prevent exposure of photosynthetic cells to artificial light on board, the carboy was covered completely with two layers of dark plastic. Samples were processed immediately after collection. The sampling depth was chosen because it reflects the surface mixed layer, and the influence of ship movement and contaminants at that depth are minimal. At selected stations (indicated in Supplementary Figure S2) additional samples were taken from the bottom of the thermocline in order to compare CO_2_ fixation rates of deeper vs surface phytoplankton communities.

### Abundance measurements and definition of regional boundaries

Concentrations of the *Synechococcus* (*Syn*) and *Pro* cyanobacteria were determined in unstained, fixed (1% paraformaldehyde, final concentration; Sigma-Aldrich, Hamburg, Germany) samples according to Olson *et al.* (1993) using a FACSort flow cytometer (Becton-Dickinson, Oxford, UK). *Pro* cells were counted in both unstained (*Pro*_unst_) and stained fixed samples (*Pro*_st_) on the basis of their red autofluorescence and 90° side light scatter, and their nucleic acid content (green fluorescence) and 90° side light scatter, respectively. Subsamples for *Pro*_st_ counting were taken from a 20 l carboy and fixed with 1% paraformaldehyde for 1 h in the dark at room temperature and stained with SYBR Green I dye (Sigma-Aldrich) (Marie *et al.*, 1997). Cellular abundances of small (<2 μm) and large (2–5 μm) plastidic eukaryotes (Plast-S and Plast-L, respectively) were determined in parallel from the same sample. Before flow cytometric analyses, a mixture of 0.5 and 1.0 μm multi-fluorescent beads (Polysciences, Eppelheim, Germany) at a calibrated concentration (Zubkov and Burkill, 2006) was added to both stained and unstained samples. The beads were used as an internal standard for calculating absolute cell concentrations and for normalising cellular red autofluorescence.

Four major oceanic regions were identified using primarily *Syn* abundances: Northern subtropical gyre (NG), equatorial waters (EQ), Southern subtropical gyre (SG) and Southern temperate waters (ST) (Hartmann *et al.*, 2012).

### Catalysed reporter deposition fluorescence *in situ* hybridisations (CARD-FISH) on flow cytometrically sorted cells

In order to confirm that the distinct, high-nucleic acid bacterial population observed by flow cytometry (Supplementary Figure S1) consists mainly of *Pro* cells, CARD-FISH hybridisations using the *Pro*-specific probe PRO405 (West *et al.*, 2001) were carried out on sorted cells at selected stations covering each province (NG, EQ and SG). The *in silico* specificity of the probe was re-evaluated by running TestProbe (part of the Silva online software packages, www.arb-silva.de; Quast *et al.*, 2013) against the Silva SSU r117 reference database. In addition, the Eubacteria-targeted probe mix Eub338I-III (Amann *et al.*, 1990; Daims *et al.*, 1999) was used to determine overall hybridisation efficiency. The details of contamination-free flow sorting of target cells and CARD-FISH are described in Gomez-Pereira *et al.* (2013). We analysed seven stations in the NG, three stations in the EQ and four stations in the SG.

### Total and cell-specific CO_2_ fixation

Before each experiment, 60 ml Pyrex glass bottles (Fisher Scientific, Loughborough, UK) were acid-soaked overnight (10% HCl) and rinsed twice with 30 ml sample sea water. After washing, 60 ml of seawater sample was added to each bottle and spiked with trace metal-clean ^14^C radiolabelled sodium bicarbonate (34.66 mM NaH^14^CO_3_; DHI, Hørsholm, Denmark). Samples were then incubated at ambient temperatures (regulated by a refrigerated water bath (Grant Instruments, Shepreth, UK)) in a 6 l water tank illuminated by a warm white light-emitting diode array (Photon Systems Instruments, Drasov, Czech Republic) adjusted to a constant output of 500 μmol photons m^−2^ s^−1^. The chosen light intensity equals half the irradiance reaching the water surface at noon-time in the equatorial region (Jitts *et al.*, 1976), because on average at 20 m depth the light intensity is reduced by 33–55%. Moreover, no photoinhibition occurs at this light intensity (Morel *et al.*, 1996). In contrast to incubations at ambient light, the constant light output made it possible to compare CO_2_ fixation rates at different stations.

Two different concentrations of NaH^14^CO_3_ were used to determine total CO_2_ fixation during a time series and to measure CO_2_ fixation of flow cytometrically sorted phytoplankton populations, respectively. Time series were carried out to ensure linear uptake of label and to guarantee that the small volumes of sorted cells are representative of the whole community (Supplementary Figure S3). Subsamples of 1.6 ml were taken at 0 and 10 h for flow cytometric analyses to ascertain that community composition remained unchanged for the duration of the experiment (Supplementary Figure S4).

For time series CO_2_ fixation measurements, 3.7 kBq ml^−1^ NaH^14^CO_3_ was added to 60 ml seawater sample. In total six Pyrex glass bottles were prepared. Five bottles were incubated for 2, 4, 6, 8 and 10 h in the light, and the remaining bottle was incubated for 10 h in the dark. At each discrete time point, the whole sample was fixed by adding 1% paraformaldehyde (final concentration) and incubated for 1 h at room temperature. Subsequently, the complete sample was filtered onto a 0.2 μm polycarbonate filter (Nuclepore, Whatman, Little Chalfont, UK), washed three times with ultra-clean water (MQ system, Millipore, Whatman, Walford, UK) and placed in a scintillation vial. Before addition of 5 ml scintillation cocktail (Goldstar, Meridian, Epsom, UK), 1 ml of 10% HCl was added, the vial gently swirled and incubated for 10–30 min to fume out non-incorporated NaH^14^CO_3_. Dark CO_2_ fixation rates were <3% of paired CO_2_ fixation rates in the light in all experiments (Supplementary Figure S3).

To determine group-specific CO_2_ fixation rates, higher NaH^14^CO_3_ concentrations had to be used because of the small size of the organisms. To 60 ml seawater sample in a Pyrex glass bottle, 246 kBq ml^−1^ NaH^14^CO_3_ was added, the sample incubated for 10 h and then fixed with 1% paraformaldehyde (final concentration). Three 1.6 ml subsamples were taken directly to determine total CO_2_ fixation and to sort *Pro*_st_. In order to sort adequate cell numbers of cyanobacteria (*Pro*_unst_ and *Syn*), 20 ml of the sample was concentrated on a 0.6 μm polycarbonate filter (Nuclepore, Whatman) mounted in a filtration unit (Swinnex, Millipore) using a syringe pump (KD Scientific, Holliston, MA, USA) at a flow rate of 2.5 ml min^−1^. This pore size was selected as it was shown in an earlier publication (Zubkov *et al.*, 1998) that the *Pro* population with visible red autofluorescence had a cell diameter of 0.63±0.03 μm. Moreover, similar 90° side light scatter values of *Pro*_unst_ before and after concentration suggest that there is no selective enrichment of larger cells because of the concentration procedure (Student's *t*-test, *P*=0.871, Supplementary Figure S5). The remaining sample was concentrated on a 0.8 μm polycarbonate filter (Nuclepore, Whatman) the same way to enrich eukaryotic phytoplankton. Apart from the 0.6 μm concentrated fraction, all samples were stained with SYBR Green I (Marie *et al.*, 1997), stored at 4 °C and sorted flow cytometrically within 10 h.

In order to determine the influence of nutrients on CO_2_ fixation, at three stations (NG and SG) a parallel incubation was carried out where 2.6 ml of nutrient-enriched sea water from 300 m depth was added to 60 ml of seawater sample from 20 m to simulate a mixing event (Supplementary Figure S2). The experiment was run in parallel to our standard 20 m incubations for 10 h under the same light regime. The sample was processed as described above for the standard incubations. The nutrient addition corresponds to an ∼20-fold increase in nitrite/nitrate concentration (0.03 and 16.83 μmol l^−1^ average ambient concentration at 20 and 300 m, respectively; Harris and Woodward, 2014) along the whole transect. At four stations (Supplementary Figure S2, EQ and SG), samples from the bottom of the thermocline were incubated in parallel at the same light intensity as surface samples to compare CO_2_ fixation rates of phytoplankton groups living at the two depths.

### Flow cytometric sorting

Different phytoplankton populations were sorted according to light scattering properties (90° or side light scatter), relative concentration of SYBR Green I stain per particle (green fluorescence; FL1, 530±30 nm), phycoerythrin content (orange fluorescence; FL2, 580±30 nm) and chlorophyll content (red fluorescence; FL3, >650 nm) using a FACSort instrument (Becton-Dickinson). Because of their low pigmentation in surface waters, we used two approaches to sort *Pro* cells. A distinct bacterial population, verified to be mainly *Pro* by CARD-FISH (>86%, Table 1), was sorted from unconcentrated, SYBR Green I stained samples (*Pro*_st_) according to side scatter and green fluorescence properties. In addition, *Pro* was sorted according to red autofluorescence from 0.6 μm concentrated unstained samples (*Pro*_unst_), as earlier studies indicated a cell diameter of >0.6 μm (Chisholm *et al.*, 1988; Vaulot *et al.*, 1990; Zubkov *et al.*, 2000). From the same sample, *Syn* cells were sorted according to their phycoerythrin content. Plast-S and Plast-L populations were sorted from 0.8 μm concentrated, stained samples using side scatter, SYBR Green I stain and red autofluorescence as defining parameters. For each population, 4–6 replicates of different cell numbers were sorted. Bacterial and eukaryotic cells were collected on 0.2 and 0.8 μm polycarbonate filters, respectively, and treated following the same procedure as for total CO_2_ measurements (see above) before counting. Radioassaying of samples was carried out using an ultra-low-level liquid scintillation counter (1220 Quantulus, Wallac, Waltham, MA, USA).

### Cell biomass estimation of *Pro* and other phytoplankton

Cell diameters of *Pro*_st_ and *Pro*_unst_ surface populations were determined on Atlantic Meridional Transect (AMT)-4 at 11 stations spanning NG, EQ and SG regions using a size fractionation method (Zubkov *et al.*, 2000). Briefly, cell concentrations were measured in unfiltered samples and filtrates after filtering samples through polycarbonate filters (Nuclepore) of different pore sizes. The filter pore size versus the percentage of cells in the corresponding filtrates relative to cell concentration in the unfiltered sample were plotted to estimate the pore size that would retain 50% of cells. That pore size was interpreted as a mean cell diameter. Average cell diameters of *Pro*_st_ (0.52±0.03 μm, *n*=30) and *Pro*_unst_ (0.6±0.05 μm, *n*=35) were significantly different (*t*-test, *P*<0.001). Mean cell biovolumes were calculated assuming a spherical cell shape. For *Pro* and *Syn* cells, conversion factors of 184 and 211 fg C μm^−3^ (Heldal *et al.*, 2003) and cell diameters of 0.52±0.03 and 0.95±0.31 μm (Zubkov *et al.*, 2000) were applied. Details of all conversion factors used in this study can be found in Table 2. A conversion factor of 200 fg C μm^−3^ (Waterbury *et al.*, 1986) was used to calculate biomass-specific CO_2_ fixation rates assuming spherical cell shape and average cell diameters of 2.0±0.1 and 3.1±0.3 μm for Plast-S and Plast-L cells, respectively (Hartmann *et al.*, 2012). These cell diameters were established for all studied regions on two consecutive AMT cruises, including AMT-20 where the here presented CO_2_ fixation rates were measured.

### Data analyses

Cell-specific CO_2_ fixation rates were determined from average per cell values of each of the sorted replicates and converted to fg C cell^−1^ h^−1^ according to (Parsons *et al.* (1984). Statistical analyses were carried out using SigmaPlot (London, UK). In case of normal distribution and equal variance, *t*-tests were carried out for comparison. If the data were nonnormally distributed or the equal variance test failed, Mann–Whitney rank-sum tests were used.

## Results

### Determination of *Pro* cell abundance and CO_2_ fixation rates

**Figure 1:**
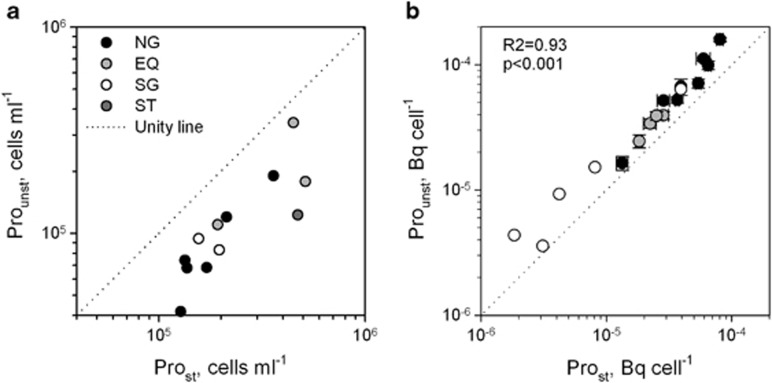
Assessment of *Prochlorococcus* abundance (**a**) and CO_2_ fixation (**b**) using either pigmentation (*Pro*_unst_) or DNA content (*Pro*_st_) to flow cytometrically separate them from other groups. Different colours indicate sampled regions in the Atlantic Ocean (EQ, equatorial region; NG, Northern Gyre; SG, Southern Gyre; ST, Southern temperate waters). Actual numbers of *Pro* are underestimated using pigmentation alone (**a**). A significant positive correlation between the two protocols suggests that *Pro*_unst_ is part of *Pro*_st_ (**b**).

Because of the low red autofluorescence of *Pro* cells in surface waters and the resulting unreliability of detection, we focussed on a distinct population within the bacterioplankton based on flow cytometrically determined cellular nucleic acid content and 90° light scatter, called *Pro*_st_ (Supplementary Figure S1; Zubkov *et al.*, 2000). The taxonomic identity of cells within this population was verified by flow sorting followed by Card-FISH using a *Pro*-targeted probe (Pro405, West *et al.* 2001) at 14 stations along the AMT (Table 1). The majority of flow sorted cells (86–94%) hybridised with the *Pro*-specific probe, corroborating the results of previous molecular studies of the same distinct population (Table 1; Zubkov *et al.*, 2007; Mary *et al.*, 2008; Gomez-Pereira *et al.*, 2013). As a control, *Pro* cells were in parallel enumerated on the basis of red autofluorescence in unstained samples (*Pro*_unst_, Supplementary Figure S1) and compared with *Pro*_st_ cell numbers revealing a significant underestimation of *Pro* abundance in *Pro*_unst_ measurements (Figure 1a).

To independently validate the molecular identification of *Pro*_st_, fixation of ^14^CO_2_ was determined in both *Pro*_st_ and *Pro*_unst_ sorted cells (Supplementary Figure S1). Both populations were photosynthetically active, and the ^14^CO_2_ fixation rate per cell of *Pro*_unst_ was 50% higher than that of *Pro*_st_ (Wilcoxon signed-rank test, *P*<0.001; Figure 1b). A strong linear correlation between the^14^CO_2_ fixation of the *Pro*_st_ and *Pro*_unst_ cells (*R*^2^=0.93, *P*<0.001) indicated that the *Pro*_unst_ cells comprised a subpopulation of the *Pro*_st_ population (Figure 1).

There could be two reasons why the *Pro*_unst_ cells systematically fixed 50% more ^14^CO_2_ than the *Pro*_st_ cells:

1. The presence of non-photosynthetic bacteria among the sorted *Pro*_st_ cells could lower the average cellular ^14^C content (because the total measured ^14^CO_2_ fixation was divided by the total number of sorted cells). The cells among the sorted *Pro*_st_ populations not hybridising with the *Pro*-targeted probe could either be *Pro* cells with ribosomal contents below detection level of FISH (for example, *Pro* cells were compromised or dead) or they could be by-sorted non-*Pro* cells displaying similar DNA fluorescence and side light scatter properties. A small proportion of sorted cells (5–6%) could not be detected by FISH with the universal bacterial probe (Table 1), and this lends support to the former explanation. However, even in the extreme (assumed) case if all probe-negative cells were by-sorted non-*Pro* cells, one could explain a discrepancy of only 6–14% between *Pro*_st_ and *Pro*_unst_ rather than the measured 50% difference.
2. On average, larger *Pro*_unst_ cells that contain proportionally higher chlorophyll amounts resulting in detectable red autofluorescence could fix more ^14^CO_2_ than smaller *Pro*_st_ cells. Indeed, cell diameter estimates carried out on an earlier AMT cruise (AMT-4) using size fractionation revealed that *Pro*_st_ cells were systematically smaller than *Pro*_unst_ cells across the Atlantic Ocean (Table 2; Zubkov *et al.*, 2000). These observations suggest that only larger or dividing *Pro* cells with higher red autofluorescence were visible above the threshold of the red fluorescence photomultiplier detector. Indeed, the abundance of *Pro*_unst_ cells was significantly lower than that of *Pro*_st_ cells (on average 58±18%, Figure 1a). Consequently, the *Pro*_unst_ cells are not representative of the entire *Pro* population in surface waters. Sorting of *Pro*_unst_ would therefore lead to overestimation of the actual cellular CO_2_ fixation rates by *Pro*, and we used hereafter *Pro*_st_ cells for more realistic measurement of CO_2_ fixation by *Pro* cells.

The *Pro* cell-specific CO_2_ fixation rates in the NG and SG were similar, but their rates were more than doubled in the EQ (*t*-test, *P*=0.002; Supplementary Figure S6 and Supplementary Table S1). The CO_2_ fixation rates of Plast-L cells followed the same pattern (*t*-test, *P*<0.001), showing increased rates in the EQ (Supplementary Figure S6 and Supplementary Table S1). In contrast, Plast-S as well as *Syn* cells showed similar rates in the NG, SG and EQ, but significantly lower CO_2_ fixation rates in the ST (*t*-test, *P*=0.042 and *P*=0.032, respectively, Supplementary Figure S6 and Supplementary Table S1).

### Comparison of cellular, red autofluorescence-normalised and biomass-specific CO_2_ fixation of *Pro*, *Syn* and small eukaryotic phytoplankton

**Figure 2:**
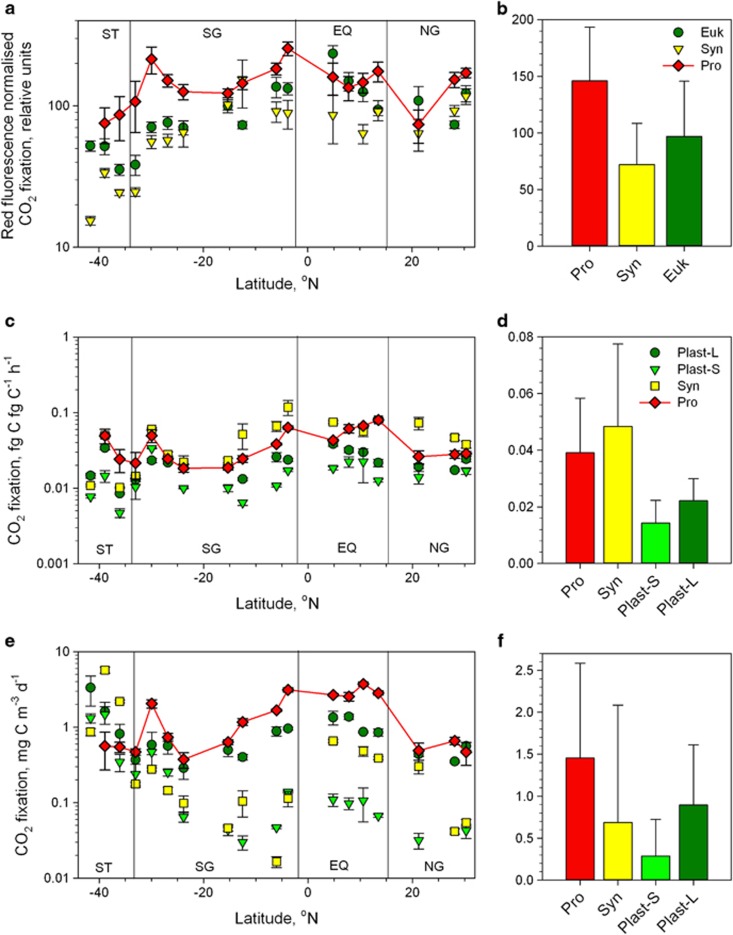
Detailed (**a**, **c**, **e**) and average (**b**, **d**, **f**) red autofluorescence-normalised, biomass-specific and population-specific CO_2_ fixation of *Pro* in comparison with *Syn* cyanobacteria and smaller and larger plastidic eukaryotes (∼2 μm, Plast-S and ∼3.1 μm, Plast-L) in the Atlantic Ocean (EQ, equatorial region; NG, Northern Gyre; ST, Southern temperate waters; SG, Southern Gyre and). Units on the y-axes are the same for (**a** and **b**, **c** and **d** and **e** and **f**). Student's *t*-test confirmed significant differences in biomass-specific CO_2_ fixation between all phytoplankton groups (*P*=0.001–0.047, see Result section for details).

Although cellular CO_2_ fixation rates positively correlated with cell sizes (*R*^2^=0.83, Supplementary Figure S6), no such relationship was found between red autofluorescence-normalised or biomass-specific CO_2_ uptake (see Table 2 for details on biomass in this study and Supplementary Table S2 for a summary table of published biomass estimates). Because it was technically impossible to determine red autofluorescence of *Pro*_st_ with required precision, red autofluorescence of *Pro*_unst_ (upper estimates, Supplementary Figures S1b and d) were used for normalisation. Consequently, the derived normalised values for *Pro* should be treated as conservative lower estimations. Red autofluorescence-normalised CO_2_ fixation shows that *Pro* despite an order of magnitude lower red autofluorescence than *Syn* (Table 2) can fix up to 4 times more CO_2_ than other small phytoplankton (*t*-test, *P⩽*0.005, Figures 2a and b) whereas CO_2_ fixation rates of *Syn* and plastidic eukaryotes are comparable.

Biomass-specific CO_2_ fixation rates of plastidic eukaryotes were significantly lower than those of cyanobacteria (Mann–Whitney, *P*<0.001) whereas *Syn* showed on average 60% higher biomass-specific CO_2_ fixation than Pro (*t*-test, *P*=0.04; Figures 2c and d). Because of the combined effect of comparatively high CO_2_ uptake rates and high abundance, *Pro* led microbial CO_2_ fixation across the low-latitude Atlantic Ocean (Mann–Whitney, *P⩽*0.038; Figures 2e and f).

**Figure 3:**
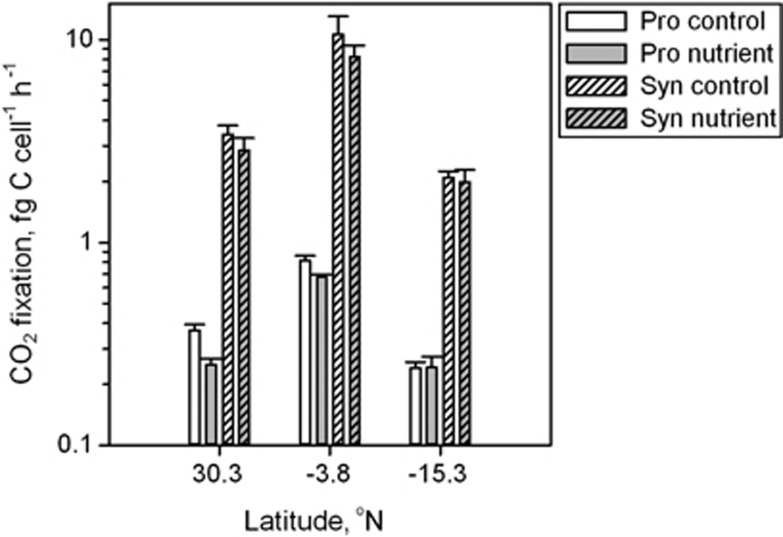
Average per cell CO_2_ fixation of surface *Pro* (empty) and *Syn* (pattern) without (white) and with addition of nutrients (grey, that is, addition of sea water from 300 m depth). NG, Northern Gyre; SG, Southern Gyre.

**Figure 4:**
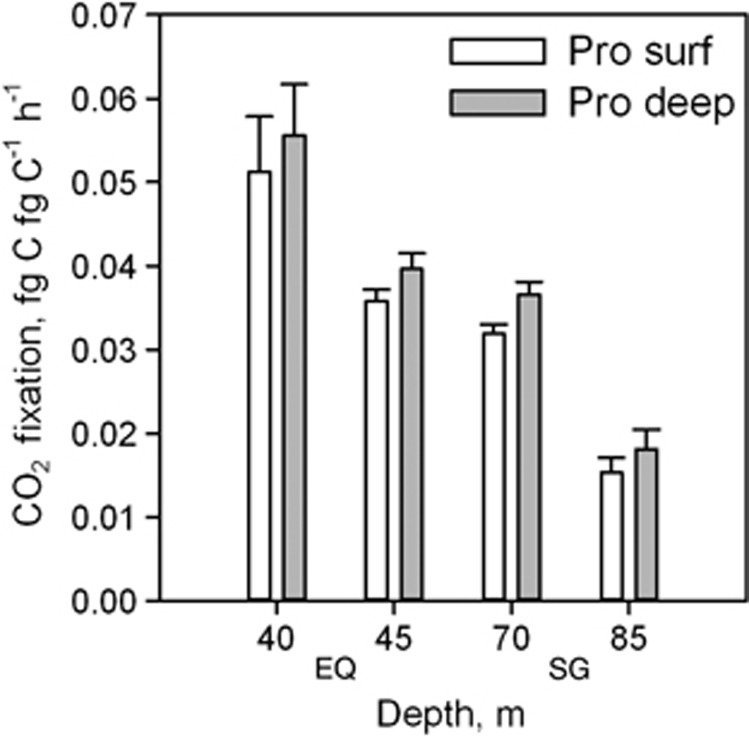
Average biomass-normalised CO_2_ fixation of *Pro* cells in surface waters (that is, 20 m depth, white) and deeper water layers (grey) in the Equatorial region (EQ) and the Southern gyre (SG). Numbers on x-axis indicate depth of the deep water sample.

To assess whether cellular CO_2_ fixation and red autofluorescence of surface *Pro* could be influenced by a lack of nutrients, additional experiments with added nutrients were performed. Nutrient addition to surface samples in the form of deep water (300 m depth) had no significant effect on CO_2_ fixation rates of either *Pro* or *Syn* cells (*t*-test, *P*>0.5, Figure 3) and did not influence red autofluorescence of *Pro* (*t*-test, *P*=0.12). Moreover, comparisons of CO_2_ fixation rates of *Pro* populations from surface waters and deeper water (bottom of the thermocline) revealed no significant differences in CO_2_ fixation rates when exposed to the same light conditions, despite stronger red fluorescence of deeper *Pro* cells (Figure 4).

## Discussion

Direct determination of group-specific CO_2_ fixation rates using ^14^C-tracer is technically challenging and has been attempted only in three other studies so far (Chisholm *et al.*, 1988; Li, 1994; Jardillier *et al.*, 2010) that were spatially restricted to small areas of the Atlantic Ocean. This new data set provides for the first time insight into CO_2_ fixation rates of four distinct phytoplankton groups across the Atlantic Ocean. Cellular CO_2_ fixation rates measured in the equatorial region are comparable to those measured in the North East Atlantic (Li, 1994; Jardillier *et al.*, 2010; Supplementary Table S3). Our slightly lower values can be most likely attributed to the differences in light regime between the studies (artificial vs ambient light). Inferred from estimates of diel synchronised cell division in the photic layer (Vaulot *et al.*, 1995) and measurements of CO_2_ fixation by *Pro* inhabiting the deeper waters (Chisholm *et al.*, 1988), *Pro* could contribute more than a half to the total CO_2_ fixation in the low-latitude Ocean. These estimates concur with our results from surface waters (Figures 2e and f).

A combination of high sunlight irradiance (up to 3000 μmol photons m^−2^ s^−1^ ;Jitts *et al.*, 1976), slow rates of vertical mixing, low inorganic nutrient availability and potential preferential grazing pressure by mixotrophic protists (Hartmann *et al.*, 2013) creates a harsh habitat for *Pro* cyanobacteria in surface waters of the low-latitude Atlantic Ocean. How do *Pro* cells remain numerous, effective CO_2_ fixers in these waters (Figure 2) with virtually undetectable red cellular autofluorescence (indicative of extremely low photosynthetic pigmentation)?

Biosynthesis of photosynthetic pigments like chlorophyll requires certain inorganic nutrients, for example, nitrogen and iron, that are depleted in the subtropical gyres, and constrained nutrient bioavailability can lead to reduced cellular pigmentation (Riemann *et al.*, 1989; Staehr *et al.*, 2002). However, both plastidic protists and *Syn* cyanobacteria can easily be detected in the same waters by their photosynthetic pigmentation, suggesting that the required nutrients are still bioavailable to those cells. Furthermore, CO_2_ fixation rates of *Pro* remained unchanged in our deep water addition experiments to simulate mixing events, suggesting that *Pro* are not nutrient limited, at least with regard to CO_2_ fixation (Figure 3). These findings are in accordance with a study in the equatorial Pacific Ocean (Vaulot *et al.*, 1995), where close to maximal (that is, nutrient unlimited) growth rates of *Pro* were estimated. In addition, *Pro* cells populating the deeper parts of the mixed layer, where nutrients are still scarce but irradiance is less intense, exhibit stronger red autofluorescence (Zubkov *et al.*, 1998).

Perhaps *Pro* cells produce only low amounts of photosynthetic pigmentation in order to achieve efficient CO_2_ fixation with minimal effort using light energy for photosynthesis as well as for photoheterotrophy, that is, redirecting a part of the collected light energy for actively importing organic molecules (Casey *et al.*, 2009; Zubkov, 2009). This would allow them to compete with *Syn* cells that spend considerable energy on the production of photoprotective pigments (Raven, 1991; Aráoz and Häder, 1999). Molecular studies of cultured *Syn* and *Pro* showed that the *Pro* response to photo-damage is modelled to minimise energy demand, for example, during high irradiance periods of the day the main metabolic processes are downregulated (Mella-Flores *et al.*, 2012). Lower biomass-specific CO_2_ fixation rates of *Pro* in comparison with *Syn* (Figures 2c and d) are, perhaps, a price worth paying to avoid constant repair of photo-damaged reaction centres in highly irradiated surface waters. That might be a reason (additional to inorganic nutrient limitation (Tarran *et al.*, 1999; Vaulot *et al.*, 1996)) for low concentrations of *Syn* cells in oligotrophic waters despite their higher biomass-specific CO_2_ fixation rates. On the other hand, the difference in biomass-specific CO_2_ fixation rates between *Pro* and *Syn* is comparatively small given the higher pigment content, up to 10 times at 1000 μmol m^−2^ s^−1^ light in cultures (Moore *et al.*, 1995) and according to red autofluorescence (Figures 2a and b) of the latter.

High red autofluorescence-normalised CO_2_ fixation by *Pro* is likely related to the high geometrical absorption cross-section owing to their small cell size (Morel *et al.*, 1993; Bailey *et al.*, 2005) and package effect that states that the light-harvesting to the effect of increased pigmentation is reduced because of a parallel decrease in the absorption cross-section (Dubinsky *et al.*, 1986; Berner *et al.*, 1989). Light harvesting efficiency of *Pro* is further increased by the unique pigment, chlorophyll a_2_ (Chisholm *et al.*, 1988; Goericke and Repeta, 1992) with absorption maximum that coincides with the wavelength of higher energy blue light. The cumulative outcome of these numerous adaptations has enabled *Pro* cells with minimal photosynthetic pigmentation to become highly efficient CO_2_ fixers (Figure 2).

## Conclusion

The results presented here demonstrate that *Pro* are highly efficient CO_2_ fixers in surface waters of the Atlantic Ocean and their red autofluorescence-normalised CO_2_ fixation rates are higher than those of *Syn* and small plastidic protists. These findings indicate that *Pro* should be specially taken into account when photosynthetic pigmentation data are used for deducing biological CO_2_ fixation in the oligotrophic open ocean.

## Figures and Tables

**Table 1:** Identification of a flow cytometrically sorted, distinct population with high-nucleic acid content as *Prochlorococcus* cyanobacteria (Pro) in different regions of the Atlantic Ocean using Pro-targeted CARD-FISH (Pro405)

*Region*	*Pro405 identification (%)*	*Eub338 identification (%)*
NG (*n*=7)	86±3	94±1
EQ (*n*=3)	94±1	95±2
SG (*n*=4)	92±2	94±2

Abbreviations: EQ, equatorial region; *n*, number of sampled stations; NG, Northern Gyre; SG, Southern Gyre.

Numbers are given as percentage of 4′,6-diamidino-2-phenylindole (DAPI)-stained cells. For comparison, percentage of positive signals for the eubacterial probe (EUB338I-III) is presented. The symbol ‘±' indicates standard error (s.e.m.).

**Table 2:** Summary table of conversion factors used to calculate red autofluorescence-normalised and biomass-specific CO_2_ fixation for the different phytoplankton populations

*Group*	*Red autofluorescence (relative units)*	*Cell diameter (μm)*	*Biovolume (μm*^*3*^)	*Carbon conversion factor (fg* *C* *μm*^*−3*^)	*Biomass (fg* *C* *cell*^*−1*^)
*Pro*_st_	NA	0.52±0.03^a^	0.07±0.004	184^b^	13.6±0.9
*Pro*_unst_	0.004±0.002	0.63±0.05^a^	0.13±0.01	184^b^	24.1±2.3
*Syn*	0.07±0.04	0.95±0.31^a^	0.45±0.15	211^b^	94.7±32.2
Euk	0.43±0.08				
Plast-S	NA	2.0±0.1^c^	4.2±0.2	200^d^	837±42
Plast-L	NA	3.1±0.3^c^	15.6±1.5	200^d^	3118±302

Abbreviations: Euk, eukaryotic phytoplankton; NA, not available; Plast-L, large (∼3.1 μm) plastidic eukaryotes; Plast-S, small (∼2 μm ) plastidic eukaryotes; *Pro*_st_, *Prochlorococcus* stained; *Pro*_unst_, *Prochlorococcus* unstained; *Syn*, *Synechococcus*.

Red autofluorescence values are based on mean red autofluorescence emitted by the individual populations and normalised to red autofluorescence of 1.0 μm multifluorescent reference beads as measured by flow cytometry. Cell biovolume was calculated on the basis of a spherical shape of the cell.

a Zubkov *et al.* (2000).

b Heldal *et al.* (2003).

c Hartmann *et al.* (2012).

d Waterbury *et al.* (1986).
