# Supplementary material for: Efficient CO2 fixation by surface Prochlorococcus in the Atlantic Ocean
Source: ISME J. 2014 Apr 24;8(11):2280–9. doi: 10.1038/ismej.2014.56 (PMC4992072; doi:10.1038/ismej.2014.56)
Supplement: Supplementary Information [file ismej201456x1.doc]

**Supplementary information**

**Efficient CO_2_ fixation by surface *Prochlorococcus* in the Atlantic Ocean**

M. Hartmann, P. Gomez-Pereira, C. Grob, M. Ostrowski, D.J. Scanlan, M.V. Zubkov

**Supplementary Table**

Table S1. Regional differences of photosynthetic microbial communities in average per cell CO_2_ fixation rates. ST=Southern temperate waters, SG=Southern Gyre, EQ=equatorial region, NG=Northern Gyre, Pro=*Prochlorococcus*, Syn=*Synechococcus*, Plast-S=small, plastidic eukaryotes (~2µm), Plast-L=large, plastidic eukaryotes (~3µm).

| **CO_2_ fixation**  **[fg C cell^-1^ h^-1^]** | **Plast-L** | **s.d.** | **Plast-S** | **s.d.** | **Pro_st_** | **s.d.** | **Syn** | **s.d.** |
| --- | --- | --- | --- | --- | --- | --- | --- | --- |
| **NG** | 63.13 | 11.06 | 12.88 | 1.72 | 0.37 | 0.02 | 4.95 | 1.71 |
| **EQ** | 94.94 | 21.45 | 15.84 | 3.89 | 0.85 | 0.21 | 6.67 | 1.18 |
| **SG** | 61.97 | 14.88 | 12.85 | 7.78 | 0.44 | 0.22 | 4.54 | 3.24 |
| **ST** | 59.46 | 41.66 | 7.50 | 4.21 | 0.50 | 0.24 | 2.24 | 2.16 |

Table S2: Published biomass (fg C cell^-1^) estimates for *Prochlorococcus*, *Synechococcus* and plastidic eukaryotes. For a more extensive evaluation of biomass estimates see Buitenhuis *et al.* (2012) and Casey *et al*. (2013)

| **Pro** | **s.d.** | **Syn** | **s.d.** | **Euk** | **s.d.** | **Reference** | **Biomass based on** |
| --- | --- | --- | --- | --- | --- | --- | --- |
|  |  |  |  | 3800 | 100 | Verity *et al.* (1992) | cultures |
| 59 |  | 250 |  |  |  | Li *et al.* (1992) | literature estimates for carbon conversion and cell diameter |
| 53 |  | 246 |  | 2108 |  | Campbell *et al.* (1994) | literature estimates for carbon conversion and cell diameter |
|  |  |  |  | 800, 1360 |  | Montagnes *et al*. (1994) | cultures |
| 49 | 9 |  |  |  |  | Cailliau *et al.* (1996) | cultures |
| 92 |  | 175 |  |  |  | Veldhuis *et al.* (1997) | cultures |
| 32 | 10 |  |  |  |  | Zubkov *et al.* (1998) | natural population |
|  |  | 350 |  |  |  | Liu *et al.* (1999) | cultures |
| 29 |  | 100 |  |  |  | Zubkov *et al*. (2000) | natural population |
| 27 | 6 |  |  |  |  | Claustre *et al.* (2002) | cultures |
|  |  |  |  | 4400 |  | Llewellyn and Gibb (2000) | cultures |
| 56 |  | 112 |  |  |  | DuRand *et al.* (2001) | cultures |
| 30 |  |  |  |  |  | Heldal *et al*. (2003) | cultures |
| 53 | 9 | 170 | 65 |  |  | Bertilsson *et al.* (2003) | cultures |
| 39 | 1 | 82 | 8 | 530 | 185 | Worden *et al.* (2004) | cultures |
| 16 | 1 | 249 | 21 |  |  | Fu *et al*. (2007) | cultures |
| 29 | 11 | 60 | 19 | 730 | 226 | Grob *et al*. (2007) | natural population |
| 6.8-73 |  | 46-94 |  |  |  | Grob *et al.* (2013) | natural population |
| 52 | 19 | 250 | 91 | 2587 | 554 | Casey et al. (2013) | natural population |
| 16^b^ and 26^a^ | 0.9^b^ and 2.2 ^a^ | 99 | 32 | 1031* | 112 | This pub | natural population |
| 41 | 21 | 163 | 92 | 2359 | 1572 | Average |  |

* weighted average of Plast-S and Plast-L

^a^ Pro_unst_

^b^ Pro_st_

Table S3: Comparison of published group-specific CO_2_ fixation rates (fg C cell^-1^ h^-1^) in surface Atlantic waters. Pro=*Prochlorococcus*, Syn=*Synechococcus*, Plast-S=small, plastidic eukaryotes (~2µm), Plast-L=large, plastidic eukaryotes (~3µm), NE=Northeast Atlantic, NG=Northern Gyre EQ=equatorial region, SG=Southern Gyre.

| **Pro** | **s.d.** | **Syn** | **s.d.** | **Plast-S** | **s.d.** | **Plast-L** | **s.d.** | **Region** | **Reference** |
| --- | --- | --- | --- | --- | --- | --- | --- | --- | --- |
| 0.81 |  | 7.68 |  |  |  | 193.16 |  | NE | Li, 1994 |
| 1.2 | 0.6 | 9.5 | 4.3 | 54.3 | 19 | 230.1 | 86.1 | NE | Jardillier *et al.* 2010 |
| 0.37 | 0.02 | 4.95 | 1.71 | 12.88 | 1.72 | 63.13 | 11.06 | NG | This study |
| 0.85 | 0.21 | 6.67 | 1.18 | 15.84 | 3.89 | 94.94 | 21.45 | EQ | This study |
| 0.44 | 0.22 | 4.54 | 3.24 | 12.85 | 7.78 | 61.97 | 14.88 | SG | This study |
| 0.50 | 0.24 | 2.24 | 2.16 | 7.50 | 4.21 | 59.46 | 41.66 | ST | This study |

**Supplementary Figures:**

Fig. S1. Flow cytometry data scatter plots of samples taken at station 74 in the South Atlantic subtropical gyre indicating Pro_st_ (a and c) and Pro_unst_ (b and d) populations at 20 m depth (a and b) and 85 m depth (c and d). No significant changes can be observed in stained samples (a and c) while there is a significant increase in red autofluorescence (FL3) observable in unstained samples (b and d). *Synechococcus* cyanobacteria (Syn) are indicated in unstained samples (b and d) for comparison.

Fig. S2. Cruise track on AMT-20 in 2010. Stations were CO_2_ fixation measurements were carried out as well as regional boundaries are indicated. Black filled circles represent stations were CO_2_ fixation at 20m was determined, nutrient addition experiments are indicated by green filled circles and blue filled circles highlight stations were in addition to 20m CO_2_ fixation was also measured for a deeper sample at the bottom of the thermocline. NG=Northern Gyre, EQ=Equatorial region, SG=Southern Gyre, ST=Southern temperate waters.

Fig. S3. Total CO_2_ fixation: (a) Selection of time series measurements proving linear ^14^C uptake during the observed time course (white and black filled circles represent dark controls, i.e. CO_2_ fixation in the absence of light), (b) Comparison of total CO_2_ fixation measured by filtering small volumes (1-3ml) or large volumes (300ml) showing comparable uptake rates in the gyre region indicating that by sampling small volumes the whole community can be captured.

Fig. S4. Abundance of different microbial organisms at the beginning (T0) and after 10 h incubation (T10) during ^14^C uptake experiments. Pro=*Prochlorococcus*, Syn=*Synechococcus*, Plast-S=small, plastidic eukaryotes (~2µm), Plast-L=large, plastidic eukaryotes (~3µm), Bpl=total bacterioplankton including heterotrophic bacteria.

Fig. S5. Comparison of 90° side light scatter geometrical means of Pro_unst_ populations before and after concentration of samples on 0.6 µm polycarbonate filter to show that SSC, indicative of cell size, remains unchanged by the procedure. The equation and R_2_ of the data’s linear regression line are presented in the graph. NG=Northern Gyre, EQ=Equatorial region, SG=Southern Gyre, ST=Southern temperate waters.

Fig S6. Detailed per cell CO_2_ fixation rates for the different microbial groups in the low latitude Atlantic Ocean. ST=Southern temperate waters, SG=Southern Gyre, EQ=equatorial region, NG=Northern Gyre, Pro=*Prochlorococcus*, Syn=*Synechococcus*, Plast-S=small, plastidic eukaryotes (~2µm), Plast-L=large, plastidic eukaryotes (~3µm).

Supplementary Fig. 1


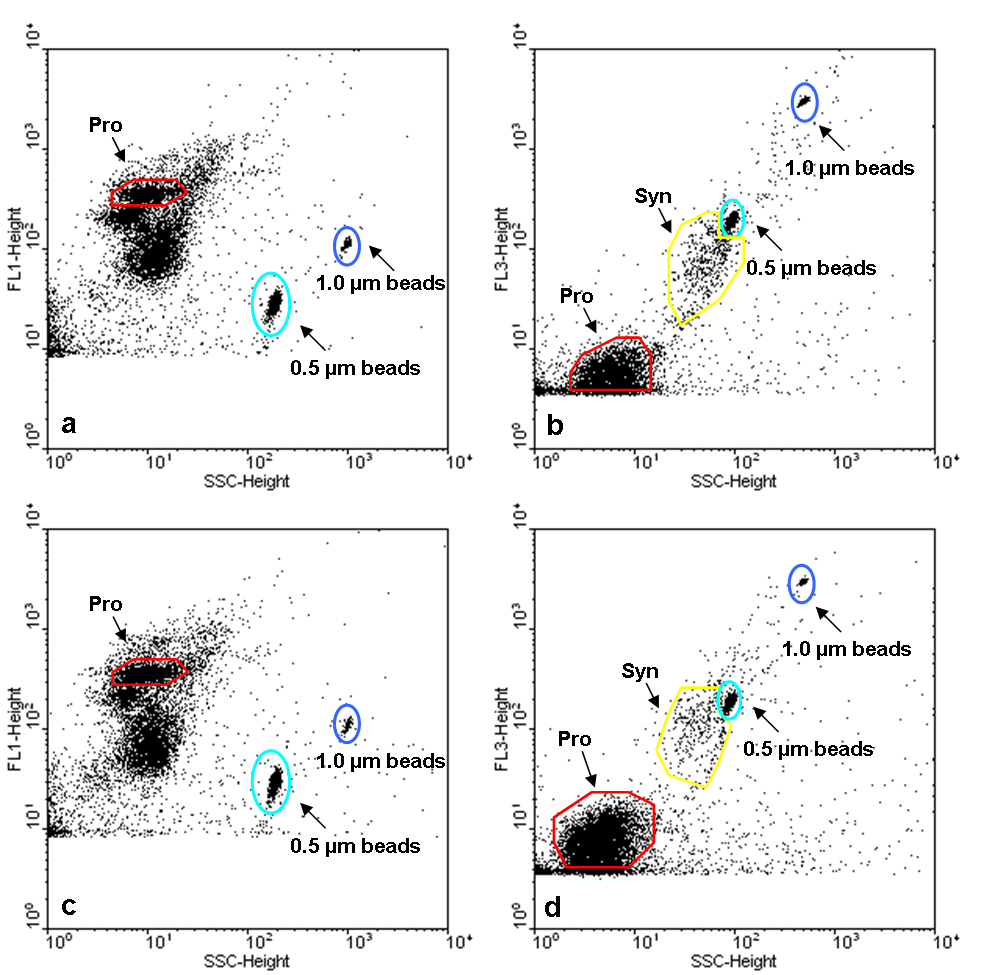


Supplementary Fig. 2


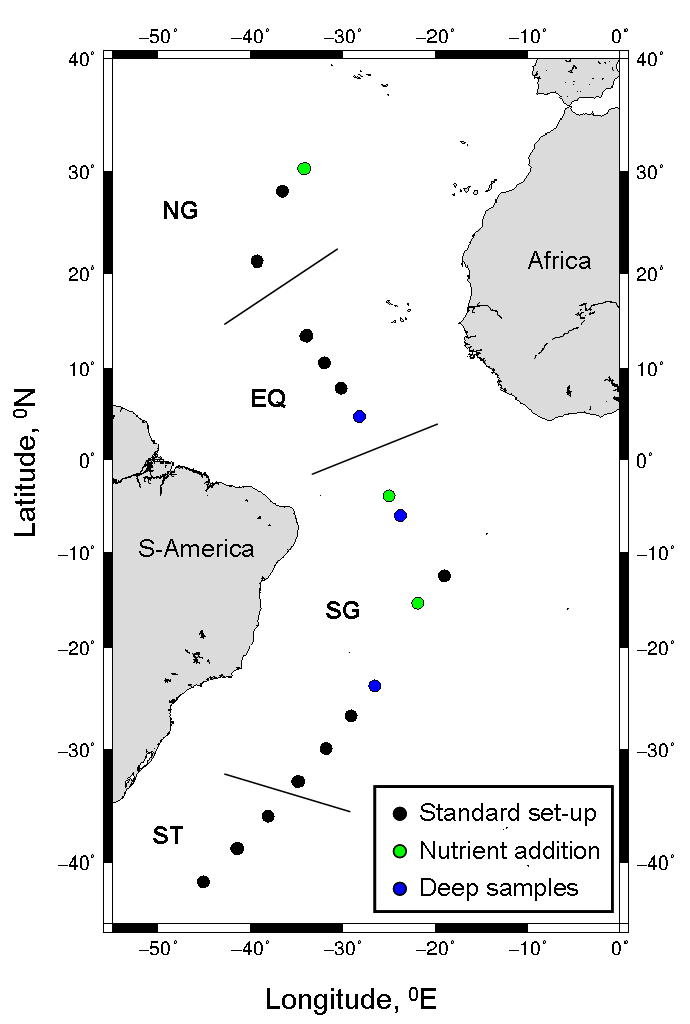


Supplementary Fig. 3


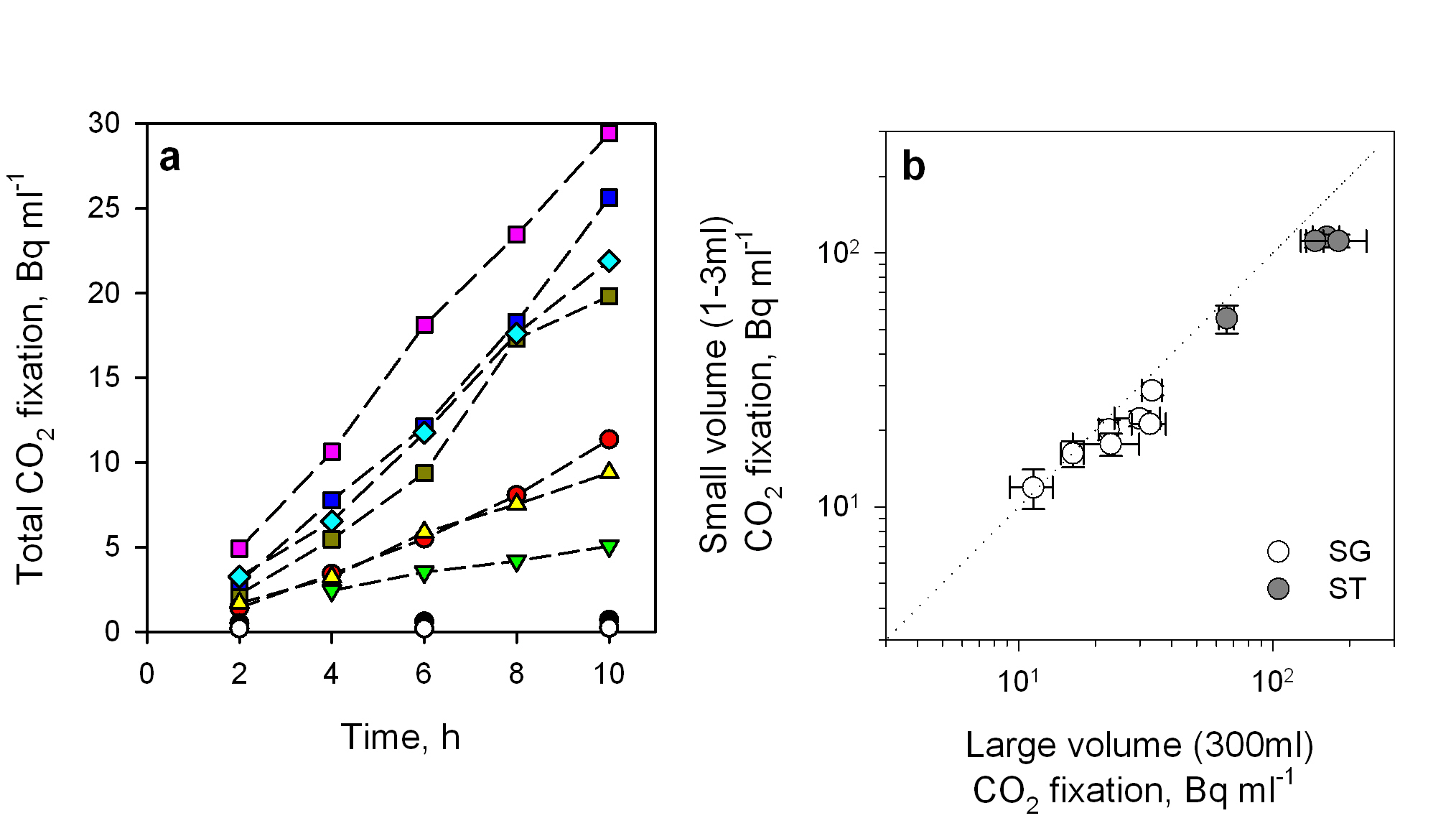


Supplementary Fig. 4


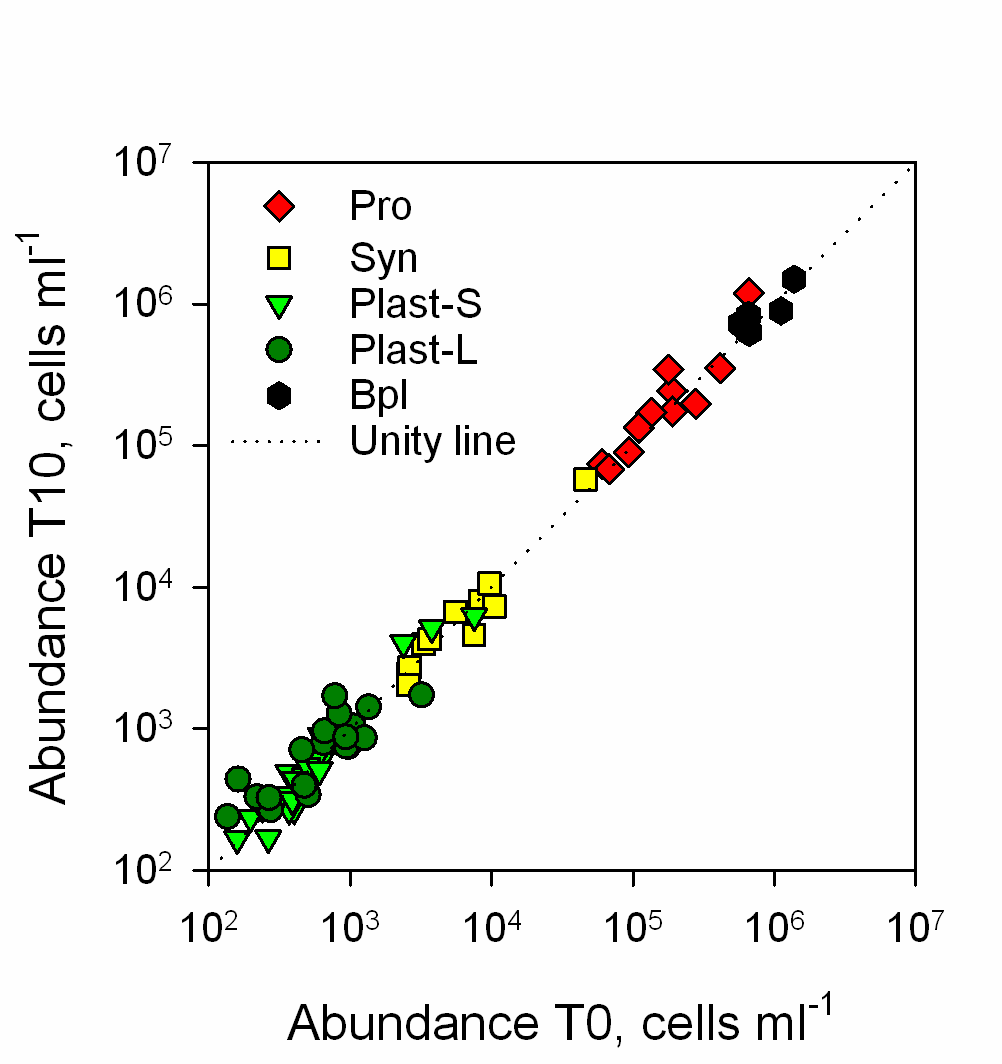


Supplementary Fig. 5


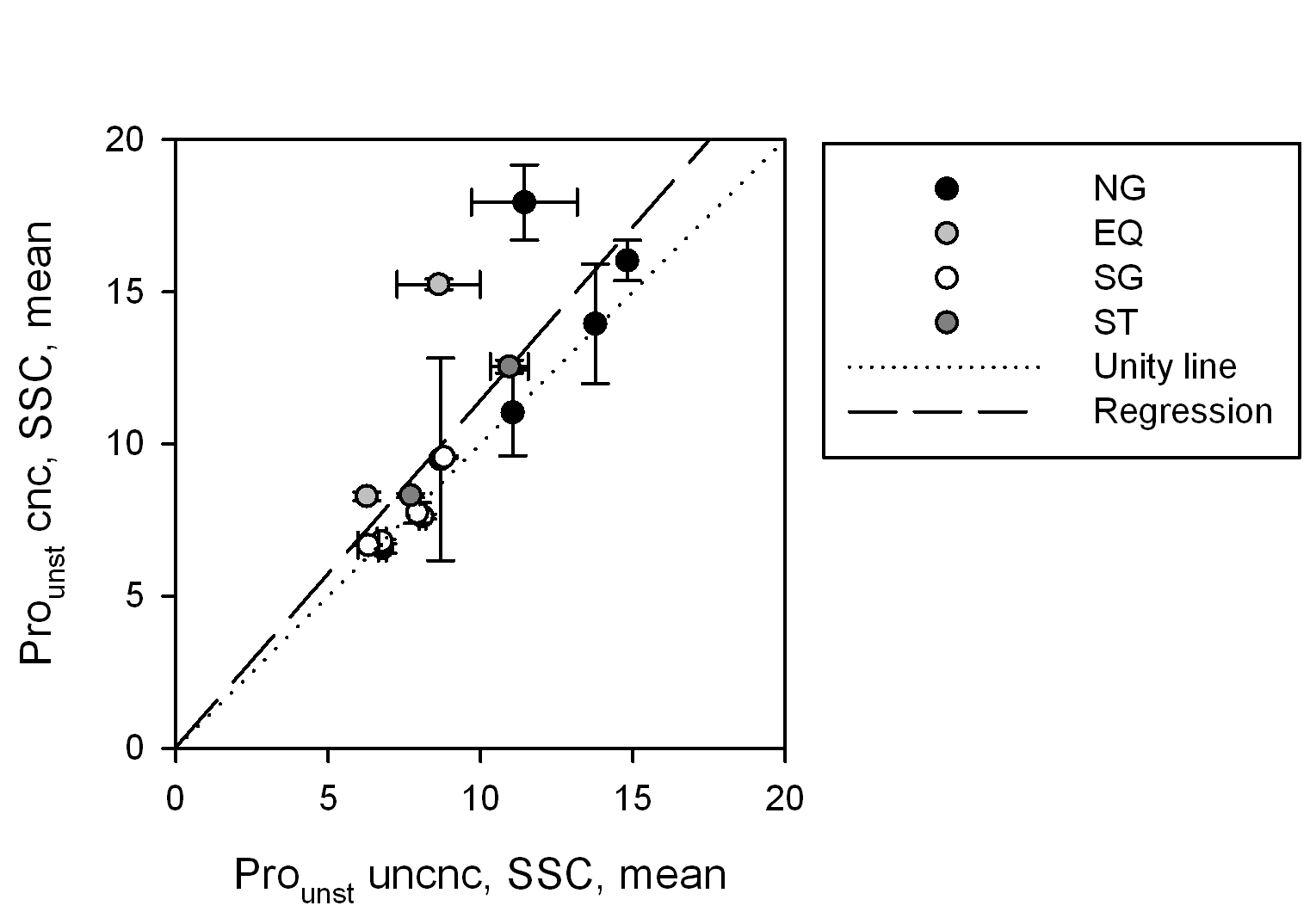


Supplementary Fig. 6


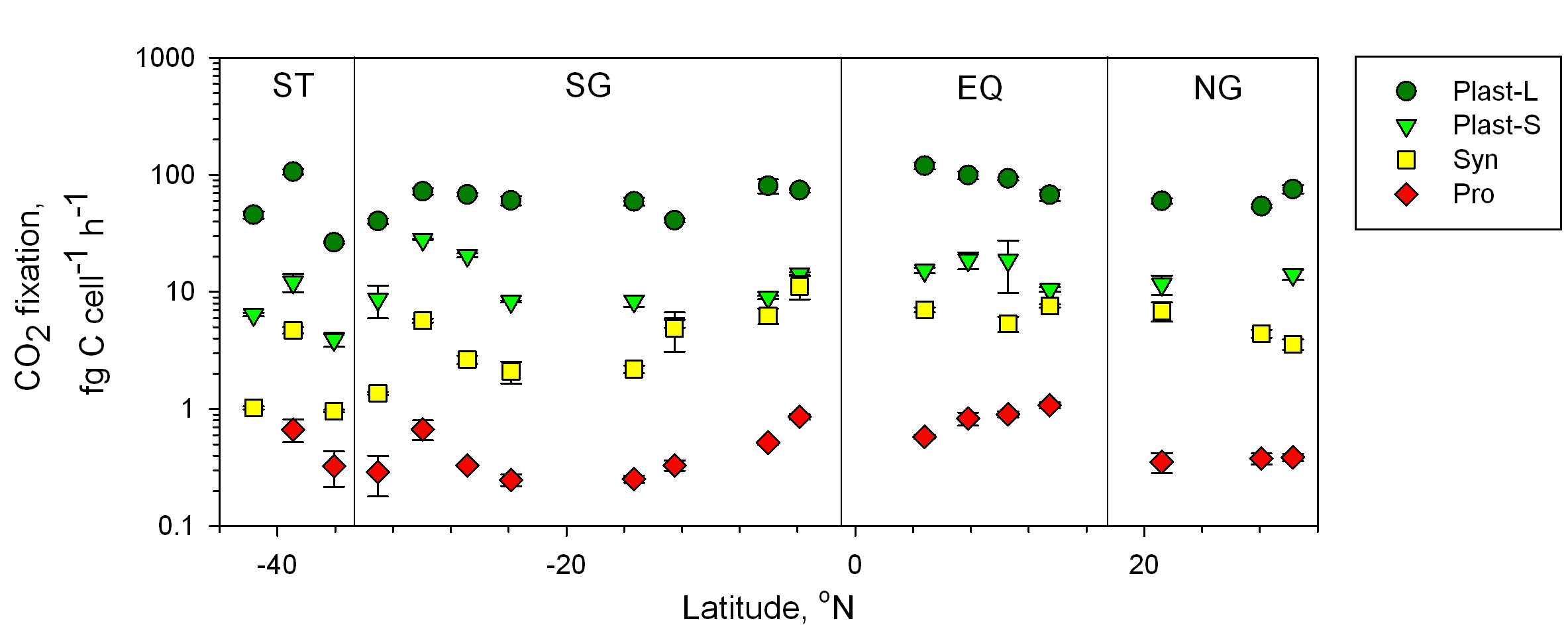


**Supplementary References**

Bertilsson S, Berglund O, Karl DM, Chisholm SW (2003). Elemental composition of marine *Prochlorococcus* and *Synechococcus*: Implications for the ecological stoichiometry of the sea. *Limnol. Oceanogr.* **48:** 1721-1731.

Buitenhuis ET, Li WKW, Vaulot D, Lomas MW, Landry MR, Partensky F *et al* (2012). Picophytoplankton biomass distribution in the global ocean. *Earth Syst. Sci. Data* **4:** 37-46.

Cailliau C, Claustre H, Vidussi F, Marie D, Vaulot D (1996). Carbon biomass, and gross growth rates as estimated from C-14 pigment labelling, during photoacclimation in Prochlorococcus CCMP 1378. *Mar. Ecol. Prog. Ser.* **145:** 209-221.

Campbell L, Nolla HA, Vaulot D (1994). The importance of *Prochlorococcus* to community structure in the central North Pacific Ocean. *Limnol. Oceanogr.* **39:** 954-961.

Casey JR, Aucan JP, Goldberg SR, Lomas MW (2013). Changes in partitioning of carbon amongst photosynthetic pico- and nano-plankton groups in the Sargasso Sea in response to changes in the North Atlantic Oscillation. *Deep-Sea Research Part Ii-Topical Studies in Oceanography* **93:** 58-70.

Claustre H, Bricaud A, Babin M, Bruyant F, Guillou L, Le Gall F *et al* (2002). Diel variations in *Prochlorococcus* optical properties. *Limnol. Oceanogr.* **47:** 1637-1647.

DuRand MD, Olson RJ, Chisholm SW (2001). Phytoplankton population dynamics at the Bermuda Atlantic Time-series station in the Sargasso Sea. *Deep-Sea Research Part Ii-Topical Studies in Oceanography* **48:** 1983-2003.

Fu FX, Warner ME, Zhang YH, Feng YY, Hutchins DA (2007). Effects of increased temperature and CO_2_ on photosynthesis, growth, and elemental ratios in marine *Synechococcus* and *Prochlorococcus* (Cyanobacteria). *J. Phycol.* **43:** 485-496.

Grob C, Ostrowski M, Holland RJ, Heldal M, Norland S, Erichsen ES *et al* (2013). Elemental composition of natural populations of key microbial groups in Atlantic waters. *Environ. Microbiol.* **15:** 3054-3064.

Grob C, Ulloa O, Claustre H, Huot Y, Alarcon G, Marie D (2007). Contribution of picoplankton to the total particulate organic carbon concentration in the eastern South Pacific. *Biogeosciences* **4:** 837-852.

Heldal M, Scanlan DJ, Norland S, Thingstad F, Mann NH (2003). Elemental composition of single cells of various strains of marine *Prochlorococcus* and *Synechococcus* using X-ray microanalysis. *Limnol. Oceanogr.* **48:** 1732-1743.

Li WKW, Dickie PM, Irwin BD, Wood AM (1992). Biomass of bacteria, cyanobacteria, prochlorophytes and photosynthetic eukaryotes in the Sargasso Sea. *Deep-Sea Research Part a-Oceanographic Research Papers* **39:** 501-519.

Liu HB, Bidigare RR, Laws E, Landry MR, Campbell L (1999). Cell cycle and physiological characteristics of *Synechococcus* (WH7803) in chemostat culture. *Mar. Ecol. Prog. Ser.* **189:** 17-25.

Llewellyn CA, Gibb SW (2000). Intra-class variability in the carbon, pigment and biomineral content of prymnesiophytes and diatoms. *Mar. Ecol. Prog. Ser.* **193:** 33-44.

Montagnes DJS, Berges JA, Harrison PJ, Taylor FJR (1994). Estimating carbon, nitrogen, protein, and chlorophyll-*a* from volume in marine phytoplankton. *Limnol. Oceanogr.* **39:** 1044-1060.

Veldhuis MJW, Kraay GW, VanBleijswijk JDL, Baars MA (1997). Seasonal and spatial variability in phytoplankton biomass, productivity and growth in the northwestern Indian Ocean: The southwest and northeast monsoon, 1992-1993. *Deep-Sea Research Part I-Oceanographic Research Papers* **44:** 425-449.

Verity PG, Robertson CY, Tronzo CR, Andrews MG, Nelson JR, Sieracki ME (1992). Relationships between cell-volume and the carbon and nitrogen content of marin photosynthetic nanoplankton. *Limnol. Oceanogr.* **37:** 1434-1446.

Worden AZ, Nolan JK, Palenik B (2004). Assessing the dynamics and ecology of marine picophytoplankton: The importance of the eukaryotic component. *Limnol. Oceanogr.* **49:** 168-179.

Zubkov MV, Sleigh MA, Burkill PH, Leakey RJG (2000). Picoplankton community structure on the Atlantic Meridional Transect: a comparison between seasons. *Prog. Oceanogr.* **45:** 369-386.

Zubkov MV, Sleigh MA, Tarran GA, Burkill PH, Leakey RJG (1998). Picoplanktonic community structure on an Atlantic transect from 50°N to 50°S. *Deep Sea Res. I (Oceanogr. Res. Pap.)* **45:** 1339-1355.
